# Bacterial colonization patterns in daily chlorhexidine care at the exit site in peritoneal dialysis patients—A prospective, randomized controlled trial

**DOI:** 10.1371/journal.pone.0184859

**Published:** 2017-10-05

**Authors:** Hsi-Hao Wang, Shih-Yuan Hung, Min-Yu Chang, Yi-Che Lee, Hsiu-Fang Lin, Tsun-Mei Lin, Su-Pen Yang, Hsi-Hsun Lin, Su-Ching Yang, Jiun-Ling Wang

**Affiliations:** 1 Internal Medicine Department, E-DA Hospital, Kaohsiung, Taiwan; 2 School of Medicine, I-Shou University, Kaohsiung, Taiwan; 3 Department of Laboratory Medicine, E-DA Hospital/I-Shou University, Kaohsiung, Taiwan; 4 Department of Nursing, National Tainan Institute of Nursing, Tainan, Taiwan; 5 Department of Internal Medicine, National Cheng Kung University Hospital, Tainan, Taiwan; Department of Medicine, National Cheng Kung University, Tainan, Taiwan; Public Library of Science, FRANCE

## Abstract

Bacterial colonization patterns in daily chlorhexidine care at the exit site in peritoneal dialysis (PD) patients were not known. We performed a prospective, randomized controlled trial enrolling 89 PD patients. After stratification by initial *Staphylococcus aureus* (SA) carrier status, patients were randomly assigned to receive daily 4% chlorhexidine care (intervention group) or normal saline (control group) at the exit site. Monthly, we cultured bacteria from the exit site and nasal swabs for 1 year. The SA colonization rates at exit site at 6 and 12 months were significantly lower in the intervention group than the control group (5.0% vs. 22.9%, p = 0.023 and 8.6% vs. 28.1%, p = 0.037 for 6 and 12 months, respectively). The Methicillin-resistant SA (MRSA) colonization rate at exit site at 6 months was similar (5.7% vs. 2.5%,p = 0.596) in control and intervention group, but significantly lower in the intervention group than the control group at exit site at 12months (0% vs. 12.5%, p = 0.047). The gram-negative bacilli (GNB) colonization rates were similar between the intervention and control groups at 6 and 12 months. Genotyping of all MRSA isolates showed ST (sequence type) 59 was the most predominant clone. In conclusion, chlorhexidine care at the exit site in PD patients may be a good strategy for SA and MRSA decolonization.

**Trial registration:** ClinicalTrials.gov NCT02446158

## Introduction

There is no consensus on what regimen is optimal for topical care of the peritoneal dialysis (PD) catheter exit site. Several methods including soap and water, povidone-iodine, hydrogen peroxide, chlorhexidine, and topical antimicrobial agents such as gentamicin or mupirocin cream have been described for care of the exit site [1, 2]. However, many of these studies were small or short-term and lacked longitudinal evaluation of bacterial decolonization efficacy. *Staphylococcus aureus* (SA) is one of most common causes of peritonitis and exit-site infection and is associated with a high PD catheter removal rate[3]. Carriers of SA had a higher rate of exit-site infection than non-carriers [4–6]. In previous studies, staphylococcal carriage prophylaxis using either mupirocin or gentimicin ointment in the nares or exit site significantly reduced the rate of exit-site infection due to SA [7–14]. However, emerging antibiotic resistance is a concern [15]. In addition, MRSA infection in PD patients is more severe than other pathogens [16]; therefore, choosing a good antiseptic for SA and/or MRSA decolonization is important.

In recent years, the use of chlorhexidine in bathing or central line dressing changes was implemented to prevent bacterial colonization and multidrug resistant bacterial infections [17–19] and was also used in hemodialysis patients [20]. Data regarding chlorhexidine used in the catheter care of PD patients are limited and it is unclear if the use of chlorhexidine for exit site care contributes to long-term bacterial decolonization and prevent for exit site infections.

## Materials and methods

### Study population and location

This randomized controlled trial (RCT) was performed from May 2010 to May 2011 (approved by local IRB NO: EMRP25098N) at E-DA Hospital, which is a 1200-bed hospital in Southern Taiwan. The total number of PD patients in E-Da hospital was 119 in May 2010. The baseline peritonitis rate at our PD center before this clinical trial in 2009 was 0.31 episode per patient-year (50% Gram-positive bacteria, 33% Gram-negative bacteria, and 17% culture negative pathogens). And the baseline exit site infection rate was 0.26 episode per patient-year (63.6% Gram-positive bacteria and 35.3% Gram-negative bacteria).

### Study ethics and informed consent

The methods were carried out in accordance with the approved guidelines. Written informed consent was obtained from all subjects. And E-DA Hospital approved the study and experimental protocols. The IRB approved us to conduct the study from Jan 1, 2010 to Dec 31, 2011. The complete date range for participant recruitment was from 2010 May 1 to 2011 May 31 and follow-up range was from 21 days to 365 days. The reason for not registering this study in ClinicalTrials.gov before enrolment of participants started is that we were not aware of trial registration and the need to register this clinical trial of infection control intervention.

### Inclusion / exclusion criteria

We enrolled patients > 20 years old who received PD for more than 3 months. Patients were excluded if they had (1) a history of psychological illness or condition that interferes with caring of a wound; (2) recent (within 1 month) exit-site infection, peritonitis, or tunnel infection; (3) recent treatment with an antibiotic administered by any route in the last month (4); or known hypersensitivity to or intolerance of chlorhexidine or mupirocin.

### Method of allocation

For allocation of the participants, an independent study nurse (who didn’t know any clinical information of all participants) allocated subjects into intervention and control groups.

### Blinding

Neither participants nor researchers were blinded to allocation. An independent nurse evaluated the exit site wound status but assessment of outcomes was not blinded due to the different color of chlorhexidine from normal saline.

### Randomization process

We used random number tables in terms of the time they enrolled into the study and stratified by nasal SA carrier status.

### Baseline clinical and microbiological assessment

We collected demographic data on underlying disease, dialysis duration, albumin levels, and SA nasal carrier status in the intervention and control groups.

### RCT intervention

The intervention group received daily cleaning of the exit site and application of 4% aqueous chlorhexidine (Antigerm Solution, Shining BioMedical Com. Ltd) with a swab. The chlorhexidine was rinsed off by normal saline after 3 min of air-drying and then gauze was applied. The control group received daily cleaning of the exit site and application of normal saline with a swab, followed by gauze. The sample size was estimated according to the following assumption. If the SA decolonization rate of 50% is assumed in intervention group and 0% is assumed in control group, at least 16 patients would be needed per group for a power of 80%. After randomization and stratified by initial SA nasal carrier status, we enrolled 50 patients in the intervention group and 39 patients in the control group.

### Intervention other than RCT intervention (nasal decolonization)

Subjects who were SA nasal carriers (two weeks before the study started) were treated with mupirocin 2% nasal ointment (bid application to both nares) and 4% chlorhexidine bath treatment for 5 days for SA nasal decolonization.

### Outcome evaluation

We performed swab cultures at the exit site and nasal site every month during follow-up at the hospital and analyzed the bacterial colonization status at 6 and 12 months as the primary outcome. An independent nurse evaluated the exit site scoring system at 6 and 12 months. The scoring systems included the following items: presence of an erythema (0, none; 1, <0.5 cm; 2, >0.5 cm), a crust (0, none; 1, <0.5 cm; 2, >0.5 cm), tenderness (0, none; 1, moderate; 2, severe), swelling (0, none; 1, moderate; 2, severe), and discharge (0, none; 1, clear; 2, purulent)[21]. The exit site infection rate was calculated and peritonitis was evaluated as a secondary outcome. An exit-site infection was defined by the presence of purulent drainage, with or without erythema of the skin at the catheter-epidermal interface. The peritonitis is defined as “an effluent cell count with white blood cells more than 100/μL (after a dwell time of at least 2 hours), with more than 50% polymorphonuclear neutrophilic cells “with or without positive gram stain or culture according to the ISPD recommendations[22]. The primary care nephrologists of the patients confirmed each clinical diagnosis in this study. If there is any sign of redness, swelling or discharge at or near the exit site found by the patients, they will call researchers or his/her healthcare providers immediately. And the study nurse will collect all information from the medical record and the patients such as adverse events, other medical procedures or recent antibiotic use during monthly follow-up.

### Bacteriology study

Clonal relationships in the MRSA isolates collected during the study period were established by pulsed-field gel electrophoresis (PFGE) and multilocus sequence typing (MLST), using the method described by Enright et al [23]. The PFGE patterns using SmaI as the restriction enzyme were analyzed using GelCompar software. Gels have been run under the same experimental conditions.

### Statistical analysis

We compared demographic data in the intervention and control groups. The means and SD were calculated for continuous variables and percentages for categorical variables. The unpaired Student's *t* test was used for comparison of the continuous variables; whereas, categorical variables were analyzed with two sided *χ*^2^ or Fisher's exact tests. The time where there was no SA and Gram-negative bacilli (GNB) colonization in the control and intervention groups were calculated using the Kaplan–Meier method. The difference between the control group versus intervention group was tested with the log-rank test. Data were analyzed with SPSS software for Windows (Release 18.0; SPSS, Chicago, IL).

## Results

**Fig 1:**
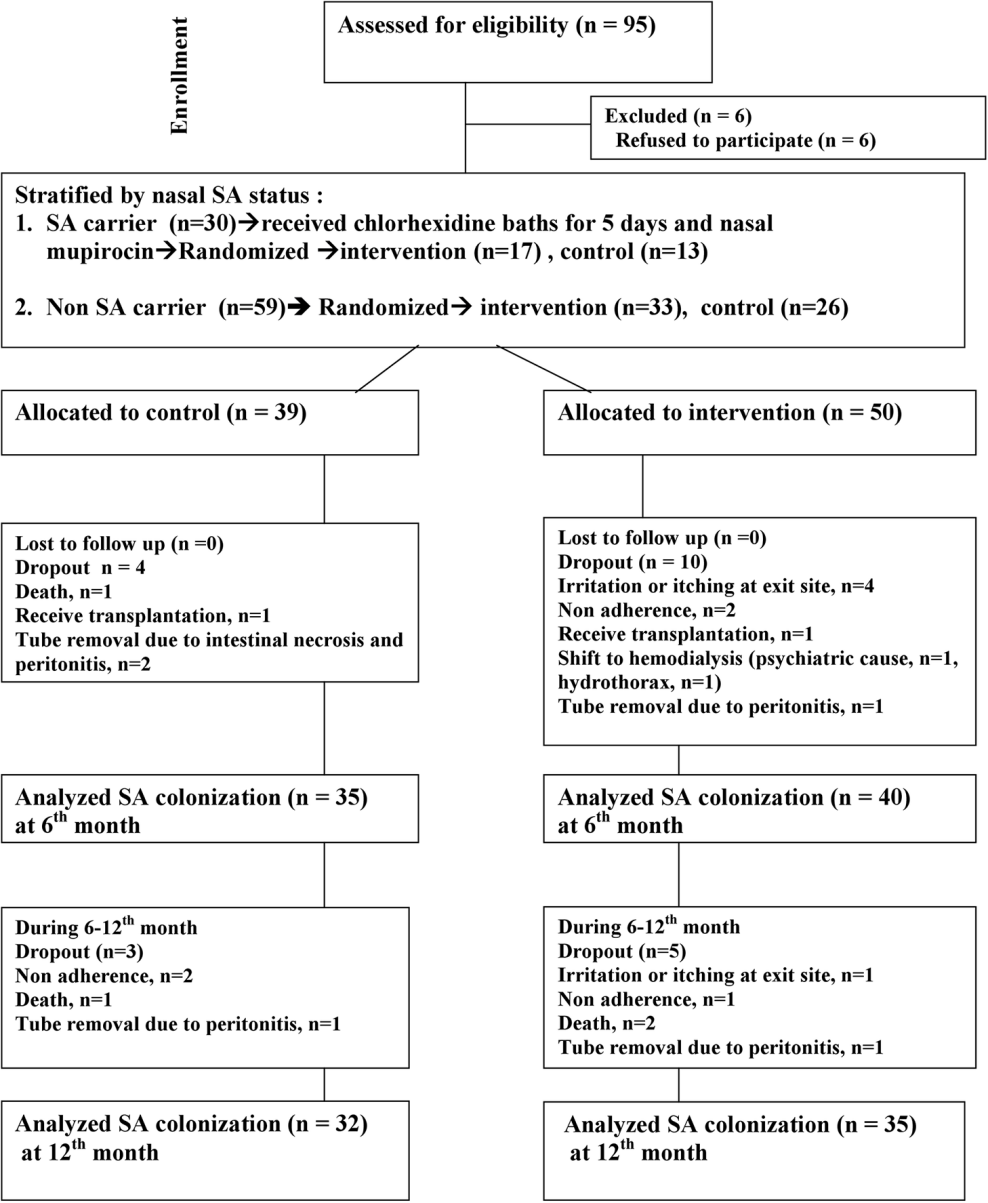
Allocation of study patients.

There were 95 patients assessed for eligibility. After excluding patients not meeting the inclusion criteria and those who declined to participate, 89 patients were allocated, as shown in Fig 1. After intervention, there were four patients intolerant of chlorhexidine due to skin itching including one patient who had focal eczema around the exit site. There were 67 patients followed up at 1 year and the reasons for dropping out are shown in Fig 1. The underlying disease, age, dialysis duration, dialysis modality (continuous ambulatory peritoneal dialysis or automated peritoneal dialysis), and baseline nasal carrier rate were similar in the control (n = 39) and intervention (n = 50) groups (Table 1). Initially, 30 nasal carriers received chlorhexidine baths for 5 days and nasal mupirocin, and 83.3% had SA decolonization in the nasal area after this procedure.

**Table 1:** Demographic data in the control and intervention groups.

Baseline	Control N = 39	Intervention N = 50	p
**Sex (Male %)**	23(59.0)	29(58.0)	0.926
**Age (mean ± SD, years)**	54.2±13.9	54.7±13.3	0.852
**Elderly (age >65 years)**	11(28.2)	11(22.0)	0.501
**Initial nasal SA colonization** ^a^	13(33.3)	17(34.0)	0.947
**Nasal SA colonization after nasal mupirocin and chlorhexidine bath eradication.**	3(7.7)	2(4.0)	0.453
**Body mass index (BMI)**	24.3±4.0	25.2±4.4	0.315
**Obesity(BMI>25)**	16(41.0)	24(48)	0.512
**Albumin level at enrollment**	3.9±0.3	3.8±0.4	0.078
**Underlying Disease**			
** Diabetes mellitus**	17(43.6)	29(58.0)	0.177
** Coronary artery disease**	5(12.8)	7(14.0)	0.872
** COPD**	1(26.0)	0(0)	0.438
** Smoking**	5(12.8)	10(20)	0.369
** History of exit site infection** ^b^	3(7.7)	9(18.0)	0.158
** History of PD peritonitis** ^b^	5(12.8)	6(12.0)	0.907
**Dialysis duration(days)**	537.5±439.3	516.0±565.0	0.845
**PD duration >1 year**	24(61.5)	24(48)	0.204
**End stage renal disease etiology**			0.339
** Hypertension**	3(7.7)	6(12)	
** Diabetes mellitus**	14(35.9)	24(48)	
** Chronic glomerulonephritis**	7(17.9)	9(18)	
** Chronic interstitial nephritis**	12(30.8)	10(20)	
** IgA nephropathy**	0(0)	1(2)	
**Dialysis modality**			
** Continuous Ambulatory Peritoneal Dialysis**	21(53.8)	25(50)	0.719
** Automated Peritoneal Dialysis**	18(46.2)	25(50)	

^a^ Before nasal mupirocin and chlorhexidine bath eradication.

^b^ More than 1 month before enrollment

The bacterial colonization patterns in these two groups are shown in Table 2.These two groups were similar in the SA and GNB colonization rates at the exit site at baseline. In the following 6–12 months, the SA colonization rate at exit site decreased in the intervention group, but increased in the control group. The SA colonization rates at 6 and 12 months were significantly lower in the intervention group than the control group at the exit site (5.0% vs. 22.9%; p = 0.023 at 6 months; 8.6% vs. 28.1%; p = 0.037 at 12 months). MRSA colonization rate at exit site at 6 months was similar (5.7% vs. 2.5%,p = 0.596) in control and intervention group. And the MRSA colonization rate at 12 months at exit site was significantly lower in the intervention group than the control group (0% vs. 12.5%; p = 0.047). There was little change in the GNB colonization during the study period in these two groups. The GNB colonization rates at the exit site at 6 and 12 months were slightly lower in the intervention group than the control group (10% vs. 14.3% at 6 months; p = 0.728; 14.3% vs. 15.6% at 12 months, p = 1.000), but did not reach statistical significance. The nasal SA colonization at baseline and 12 months was similar in these two groups; although, the SA colonization was lower in the intervention group at 6 months (17.5% vs. 37.1%, p = 0.055). Exit site scoring was worse in the control group than the intervention group at 6 months (0.66 vs. 0.12, p = 0.008), but was similar at 12 months (0.42 vs. 0.45, p = 0.88) (Table 2).

**Table 2:** Nasal and exit site SA/GNB colonization rates and wound scores at 1^st^ month and 6^th^ and 12^th^ months.

	Control N = 39	Intervention N = 50	p
**1**^**st**^ **month**			
** Nasal SA colonization**	3(7.7)	2(4.0)	0.453
** Exit site SA colonization**	6(15.4)	5(10)	0.444
** Exit site MRSA colonization**	2(5.1)	0(0)	0.189
** Exit site GNB colonization** ^a^	5(12.8)	5(10.0)	0.854
** Exit site score**	0.41±0.64	0.40±0.61	0.938
**6**^**th**^ **months**	N = 35	N = 40	
** Nasal SA colonization**	13(37.1)	7(17.5)	0.055
** Exit site SA colonization**	8(22.9)	2(5.0)	0.038
** Exit site MRSA colonization**	2(5.7)	1(2.5)	0.596
** Exit site GNB colonization** ^b^	5(14.3)	4(10)	0.728
** Exit site score**	0.66±1.19	0.12±0.40	0.008
**12**^**th**^ **months**	N = 32	N = 35	
** Nasal SA colonization**	8(25)	10(28.6)	0.742
** Exit site SA colonization**	9(28.1)	3(8.6)	0.037
** Exit site MRSA colonization**	4(12.5)	0(0)	0.047
** Exit site GNB colonization** ^c^	5(15.6)	5(14.3)	1.000
** Exit site score**	0.42±0.83	0.46±0.95	0.880

^*a*^. GNB: Gram negative bacilli and some cases have more than 2 gram negative pathogens. Including *Pseudomonas aeruginosa* (n = 4), *Escherichia coli* (n = 2), *Proteus mirabilis* (n = 1), *Chryseobacterium indologenes* (n = 1), *Enterobacter cloacae* (n = 1), *Acinetobacter lwoffii* (n = 1), *Acinetobacter junii* (n = 1).

^*b*^. *P*. *aeruginosa* (n = 2), *Serratia marcescens* (n = 1), *Klebsiella pneumoniae* (n = 1), *E*. *coli* (n = 1), *P*. *mirabilis* (n = 1), *C*. *indologenes* (n = 1), *Citrobacter diversus* (n = 1)

^*c*^. *P*. *aeruginosa* (n = 4), *Citrobacter diversus* (n = 3), *Serratia marcescens* (n = 1), *E*. *coli* (n = 1), *Acinetobacter calcoaceticus* (n = 1), *Acinetobacter*. *lwoffii* (n = 1)

**Fig 2:**
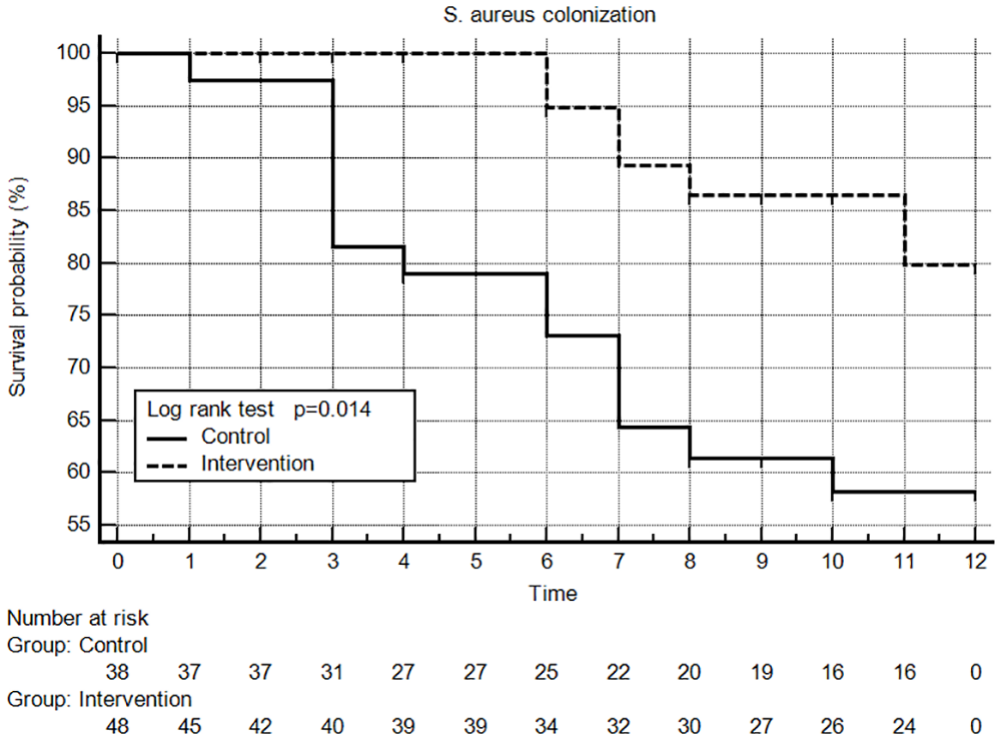
Kaplan Meir plot of SA colonization at the exit site.

**Fig 3:**
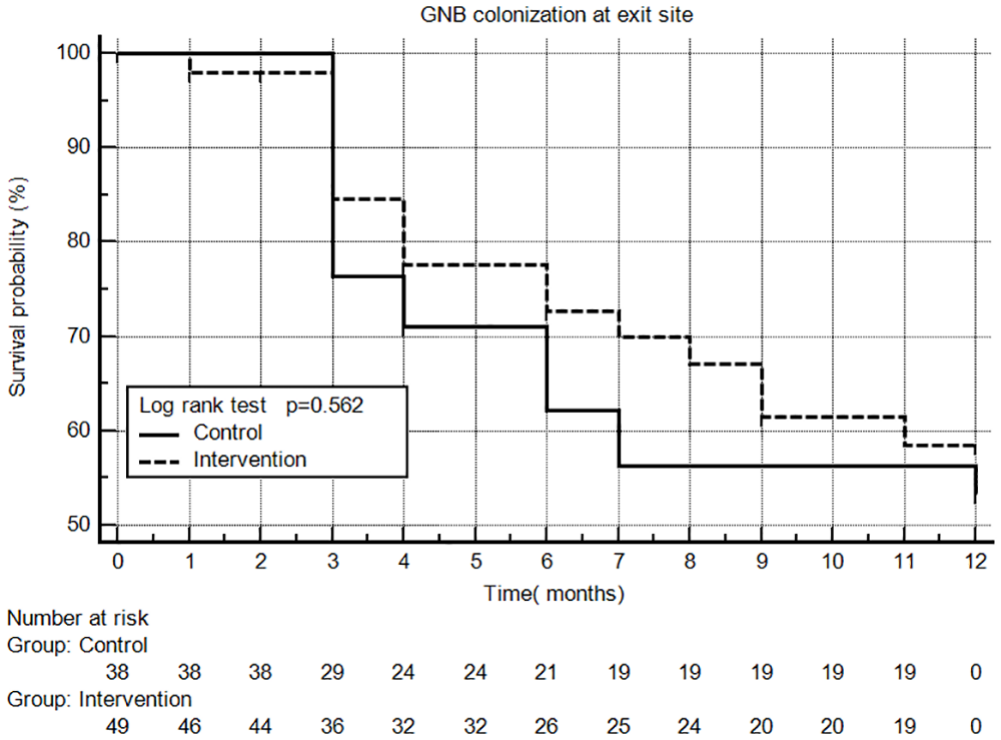
Kaplan Meir plot of GNB colonization at the exit site.

Kaplan–Meier plots of the proportion of cases without bacterial colonization are shown in Figs 2 and 3. The intervention group was free of SA colonization longer than the control group (log-rank test, p = 0.014), but there was no significant difference in the time without GNB colonization (Log-rank test, p = 0.562) in these two groups. As shown in Table 3, the exit site infection rate was slightly higher in the control group than the intervention group (one episode every 40 months vs. one episode every 80 months; p = 0.392), and the rate of peritonitis was slightly lower in the control group than the intervention group (one episode every 46 months vs. one episode every 17 months; p = 0.202); although, these differences did not reach statistically significant.

**Table 3:** Pathogen distribution in exit site infections and peritonitis.

Pathogen	Control N = 39	Case N = 50	p
**Exit site infection number**	N = 4	N = 4	
**Exit site infection rate per year**	0.30	0.15	0.392
** *S*. *aureus*** ^a^	3^a^ (7.7)	1(2.0)	0.315
** Other pathogens**			
** *P*. *aeruginosa***	1(2.6)	1(2.0)	
** *S*. *marcescens***	0(0)	1(2.0)	
** Non-growth**	0(0)	1(2.0)	
**Peritonitis number**	N = 3	N = 11	
**Peritonitis rate per year**	0.26	0.72	0.202
** *S*. *aureus***	1(2.6)	2(4.0)	1.000
** Other pathogens**			
** Coagulase negative staphylococci**	0(0)	2(4.0)	
** Viridans streptococcus**	0(0)	2(4.0)	
** GNB**	2(5.2)	4(8.0)	
** Non-growth**	0(0)	1(2.0)	

^a^ MRSA (n = 1)

**Fig 4:**
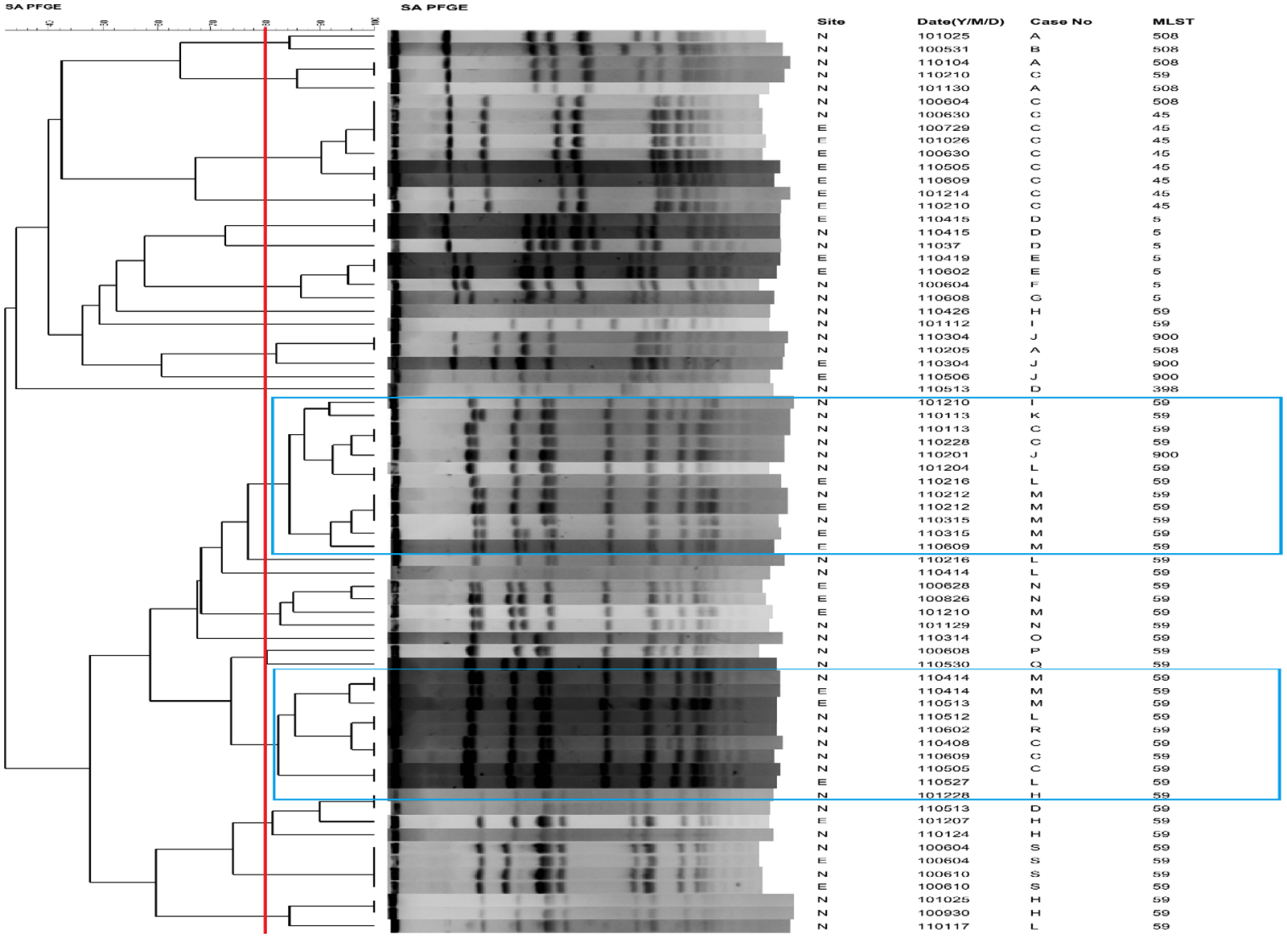
The PFGE dendrogram with molecular characterization for nasal and exit site carriage MRSA isolates. The PFGE cluster was assigned to isolates having 80% or greater similarity from the dendrograms. The blue line indicates the major pulsotype. Cropped gels have been run under the same experimental conditions. Multi-locus sequence typing (MLST). Site: N: nasal; E: exit site.

As shown in Table 3, the exit site infection rate was 0.30 episode per year in the control group and 0.15 episode per year the intervention group. And the differences in the peritonitis rate between the two groups did not reach statistical significance. During the study period, there were 19 cases positive for MRSA from the nasal swab of PD patients (Fig 4, Case A to Case S). In these MRSA isolates collected from nasal swab, the most common MLST type was ST59 (68.4%), followed by ST5 (15.8%), and other ST types included ST45, ST398, ST508, and ST 900. From the PD exit site swab, there were eight cases who were MRSA positive, and the most common type was ST59 (50%). From the PFGE dendrogram, the two major pulsotypes were ST59, and some patients shared the same pulsotype (Fig 4).

## Discussion

This is the first study to evaluate the efficacy of SA decolonization after chlorhexidine care at the exit site in PD patients over 1 year. The baseline microbiology distribution at exit site colonization and subsequent exit site infection rate and peritonitis rate in this study were similar to the literature [14, 22, 24, 25]. There was 8% of skin irritation in chlorhexidine users. The results showed the effectiveness in SA and MRSA decolonization, but not GNB decolonization at 6 and 12 months. The efficacy in preventing exit site infections was not obvious. The MRSA isolates collected in this study were mostly the CA-MRSA strain (ST59).

Chlorhexidine and iodine have been recommended as cleansing agents for use in PD care in the International Society of Peritoneal Dialysis (ISPD) guidelines [26]. An update Cochrane review including 39 studies concluded topical disinfectant use compared with standard care or other active treatment had uncertain effects to reduce exit-site/tunnel infection or peritonitis[27].Concerning about the possible cytotoxic effect, povidone-iodine was not the routinely used as cleansing agent for long term exit site care in our PD center [22]. Lee et al [28]compared the usefulness of normal saline for exit-site care with that of povidone–iodine and they found no significant differences in the incidences of exit-site infection and peritonitis but the incidences of skin irritation and itching were higher in the povidone–iodine group. There are no RCTS establishing the superiority of chlorhexidine over normal saline or povidone-iodine for exit site care in PD patients. A literature review identified three studies of a chlorhexidine base regimen for PD catheter exit site care without longitudinal bacterial colonization data.[29–31] One study compared the effectiveness of chlorhexidine versus liquid soap and showed the exit site infection rate was lower with chlorhexidine use.[29] Another study compared povidone-iodine versus chlorhexidine and sodium hypochlorite care at peritoneal catheter exit site and found no difference among these groups.[30]

The ISPD also suggests the use of mupirocin and gentamicin after cleaning for PD patients [22, 26]. In a RCT, gentamicin ointment helped prevent GNB exit site infections [8]. Our data showed chlorhexidine was not as effective for GNB decolonization as it was for SA decolonization. Bacterial colonization was significantly reduced in patients with central vascular lines who received chlorhexidine gluconate versus povidone-iodine for insertion-site skin disinfection[18]. However, some *Pseudomonas* species and gram-negative organisms have high-levels of resistance to chlorhexidine [32–36]. One study showed good pseudomonas survival in the biofilm under 4% Chlorhexidine [35]. In contrast with MRSA, there is little evidence that chlorhexidine bathing reduces carriage of or infections with GNB [19]. A neonatal skin decolonization study also showed a greater effect on SA than GNB [37, 38]. Current chlorhexidine bathing studies did not show solid evidence for decolonization of GNB. Bernardini et al showed gentamicin ointment was better at preventing a GNB infection in PD patients [11]. Further studies are necessary to evaluate the use of gentamicin cream after chlorhexidine cleaning.

There is no systemic and longitudinal surveillance of molecular epidemiology in MRSA isolates from PD patients. The MRSA isolates collected in this study were mostly the community acquired-MRSA strain (ST59), which is the most predominant community acquired-MRSA strain in Taiwan and also the predominant nasal colonization strain in hemodialysis patients [39, 40]. This implies most of the MRSA isolates in PD patients had a community origin. From the PFGE, the same pulsotype was found among different patients, indicating possible cross transmission between patients. This implies MRSA colonization may come from family members or other common source such as healthcare worker. In addition to antiseptic use in PD catheter care, hand hygiene is also important [22].

This study has some limitations. First, the number of subjects may be too low to evaluate the effect of preventing exit site infections and the analyses did not have multiple comparison adjustment. Second, the healthcare workers or patients were not blinded because of the difference in the color of the antiseptic solutions. Third, there is an increase of GNB colonization at the exit site and increased peritonitis rate (11 vs. 3) is seen in the intervention group compared to control though both did not reach statistical significance. Gram negative bacteria are the most prevalent organisms in peritonitis and yet chlorhexidine does not effectively decolonize against these organisms. We do not know if this may be due to low number of subjects in the study or this is because chlorhexidine is selecting out for the most prevalent organism. On the other hand, increased peritonitis rate in intervention group led to more frequent antibiotics exposure and it may have an impact on exit site colonization and infection. And although we list some important baseline data in two groups, some of peritonitis risk factors such as constipation, hypokalemia, connection method, medical procedures, and technique error were not reported. And this may lead to some confounding in peritonitis rate, however, the impact on the exit site colonization pattern or infection may be minimal. Back to the real world, although daily chlorhexidine exit site intervention had no obvious efficacy in preventing exit site infections in this study. The intervention did reduce the exit site infection trend in our PD center from 2009 to 2011 (exit site infection rate: 0.26, 0.18, and 0.09 episodes per patient-year, respectively). However, the trend of peritonitis rate reduction was not so obvious (unpublished data from E-DA Hospital). Since we only evaluated nasal decolonization in the first month, we don’t know if further re-colonization in the nasal area led to cross contamination at the exit site.

In conclusion, chlorhexidine care at the exit site in PD patients may be a good strategy for SA and MRSA decolonization. However, the efficacy of chlorhexidine in GNB decolonization and prevention of exit site infection and peritonitis is not clear.

## Supporting information

S1 File Consort. (DOCX)

S2 File For data sharing. (SAV)

S3 File Protocol. (PDF)

S4 File Protocol in Chinese. (DOCX)
