# Supplementary material for: Bacterial colonization patterns in daily chlorhexidine care at the exit site in peritoneal dialysis patients—A prospective, randomized controlled trial
Source: PLoS One. 2017 Oct 5;12(10):e0184859. doi: 10.1371/journal.pone.0184859 (PMC5628800; doi:10.1371/journal.pone.0184859)
Supplement: S3 File — (PDF) [file pone.0184859.s003.pdf]

Purpose

Chlorhexidine is used in central line dressing changes and is effective in reducing line infections. It is unclear if daily chlorhexidine care at the exit site in peritoneal dialysis (PD) patients can reduce the risk of Staphylococcus aureus (SA) colonization or exit site infection.

Condition	Intervention	Phase
Peritoneal Dialysis Catheter Exit Site Infection	Drug: Chlorhexidine gluconate	Phase 4

Study Type: Interventional

Study Design: Allocation:Randomized
EndpointClassification:Safety/EfficacyStudy
InterventionModel:ParallelAssignment
Masking:OpenLabel
PrimaryPurpose:Prevention

Official Title: A Randomized Trial of Daily Chlorhexidine Care at the Exit Site in Peritoneal Dialysis Patients for Bacterial Decolonization and Prevention of Infection

Primary Outcome Measures:

- exit site bacterial colonization status [TimeFrame:1 year] [Designatedassafetyissue:Yes]

  We performed swab cultures at the exit site and nasal site every month during follow-up at the hospital and analyzed the bacterial colonization status at 6 and 12 months as the primary outcome.

Secondary Outcome Measures:

- The exit-site infection rate [TimeFrame:1 year] [Designatedassafetyissue:Yes]

An exit-site infection was defined by the presence of purulent drainage, with or without erythema of the skin at the catheter-epidermal interface.

Enrollment: 89

Study Start Date: May 2010

Study Completion Date: May 2011

Primary Completion Date: May 2011 (Final data collection date for primary outcome measure)

Arms	Assigned Interventions
Experimental: Chlorhexidine gluconate<br>PD (peritoneal dialysis) paitents with daily chlorhexidine exit site care	Drug: Chlorhexidine gluconate<br>The intervention group received daily cleaning of the exit site and application of 4% chlorhexidine (Antigerm Solution, Shining BioMedical Com. Ltd) with a swab. The chlorhexidine was rinsed off after 3 min of air drying and then gauze was applied.<br>Other Name: Chlorhexidine
No Intervention: Control group<br>PD (peritoneal dialysis) patients with usual (Normal saline) exit site care	

Detailed Description:

There is no consensus on what regimen is optimal for topical care of the peritoneal dialysis (PD) catheter exit site. Several methods including soap and water, povidone-iodine, hydrogen peroxide, chlorhexidine, and topical antimicrobial agents such as gentamicin or mupirocin cream have been described for care of the exit site. However, many of these studies were small or short-term and lacked longitudinal evaluation of bacterial decolonization efficacy. Staphylococcus aureus (SA) is one of most common causes of

peritonitis and exit-site infection and is associated with a high PD catheter removal rate. Carriers of SA had a higher rate of exit-site infection than non-carriers. In previous studies, staphylococcal carriage prophylaxis using either mupirocin or gentimicin ointment in the nares or exit site significantly reduced the rate of exit-site infection due to SA. However, emerging antibiotic resistance is a concern. In addition, MRSA infection in PD patients is more severe than other pathogens; therefore, choosing a good antiseptic for SA and/or MRSA decolonization is important.

In recent years, the use of chlorhexidine in bathing or central line dressing changes was implemented to prevent bacterial colonization and multidrug resistant bacterial infections and was also used in hemodialysis patients. Data regarding chlorhexidine used in the catheter care of PD patients are limited and it is unclear if the use of chlorhexidine for exit site care contributes to long-term bacterial decolonization and acts as a prophylaxis for exit site infections.

Eligibility

Ages Eligible for Study:	20 Years and older
Genders Eligible for Study:	Both
Accepts Healthy Volunteers:	No

Criteria

Inclusion Criteria:

- patients > 20 years old who received PD for more than 3 months

Exclusion Criteria:

- a history of psychological illness or condition that interferes with caring of a wound
- recent (within 1 month) exit-site infection, peritonitis, or tunnel infection
- recent treatment with an antibiotic administered by any route in the last month
- or known hypersensitivity to or intolerance of chlorhexidine or mupirocin
