# Supplementary material for: Bacterial colonization patterns in daily chlorhexidine care at the exit site in peritoneal dialysis patients—A prospective, randomized controlled trial
Source: PLoS One. 2017 Oct 5;12(10):e0184859. doi: 10.1371/journal.pone.0184859 (PMC5628800; doi:10.1371/journal.pone.0184859)
Supplement: S4 File — (DOCX) [file pone.0184859.s004.docx]

**財團法人義大醫院**

**臨床試驗計畫書**

計畫名稱：使用消毒洗手液(Chlorhexidine)清潔腹膜透析導管出口是否有助於降低腹膜透析患者導管出口處感染之機會?

**財團法人義大醫院人體試驗計畫書目錄**

| 內 容 | 頁碼 |
| --- | --- |
| 1. 計畫書內容 |  |
| 1.摘要 |  |
| 2.試驗主題 |  |
| 3.試驗目的 |  |
| 4.試驗方法 |  |
| （1）接受試驗者標準及數目 |  |
| （2）試驗設計及進行方法 |  |
| （3）試驗期限及進度 |  |
| （4）追蹤或復健計畫 |  |
| （5）評估及統計方法 |  |
| 5.預期試驗效果 |  |
| 6.可能傷害及處理 |  |
| 7.有關文獻報告及其證明文件 |  |
| 8.所需藥品或儀器設備，包括必須進口之藥品或儀器名稱、數量。 |  |

**財團法人義大醫院人體試驗計畫書**

執行期限：自 年 月起 至 年 月止

| 填寫說明：  1.請依各項內容依序填寫。  2.填寫文字以中文為主，必要時加以英文。  3.計畫內容有引據或應用文獻者，應分別加註其出處。  4.本文件格式可自動延伸 | |
| --- | --- |
| 主持人：王曦澔 | 執行單位：腎臟科 |
| 協同主持人：王峻令、洪士元、張敏育、李宜哲、何立鈞、賴重旭 | |
| 試驗名稱：每日使用消毒洗手液(Chlorhexidine)清潔腹膜透析導管出口是否有助於降低腹膜透析患者導管出口處感染之機會? | |
| 摘要：反覆或嚴重之腹膜透析(Peritoneal Dialysis)導管出口處感染(Exit Site Infection)或腹膜炎(Peritonitis)，經常是導致腹膜透析患者住院、被迫移除導管、放棄腹膜透析甚至死亡之重要原因。根據統計顯示，導管出口處之感染將會增加腹膜透析患者未來發生腹膜炎之機會(增加六倍之風險)。因此，如何降低導管出口處感染之機會，是許多腎臟科及感染科醫師共同研究的目標。金黃色葡萄球菌(*Satphylococcus aureus*)是最常導致導管出口感染或腹膜炎的菌種，也是造成患者被迫移除導管主要原因。依過去的研究資料顯示，鼻腔金黃色葡萄球菌(*Satphylococcus aureus*)帶菌之腹膜透析患者,相較於不帶菌之患者，有更高的比例會發生導管出口感染以及腹膜炎，因而，國際腹膜透析醫學會(ISPD)於2005年提出建議，針對鼻腔金黃色葡萄球菌帶菌之腹膜透析患者，可給予Mupirocin 2% oint塗抹於鼻腔治療，然而，長期使用Mupirocin後續引發之抗藥性問題目前仍令許多專家學者擔心。目前，有越來越多的證據顯示，利用感染源控制(Source Control)的方式，可預防住院患者發生院內感染，其中利用Chlohexidine幫助患者清潔洗澡已被證實具有良好感染源控制的效果。Chlohexidine消毒洗手液本身亦是國際腹膜透析醫學會所同意可用來做為導管出口照護之藥品，但目前尚無針對利用Chlohexidine消毒洗手液作為腹膜透析患者感染源控制之相關研究，故提出本研究計劃，希望比較加強鼻部及導管出口處感源控制之方式與傳統日常照護方式，兩者之間是否有具體降低腹膜透析患者感染之差異性。  Repeat or severe exit site infection(ESI) and peritonitis are the major complications of peritoneal dialysis, leading to Tenckhoff catheter removal, technique failure, hospitalization and increased mortality. Exit-site infections are associated with a substantially increased risk of subsequent peritonitis (up to six fold). *Staphylococcus aureus* is the most common cause of ESI (25% – 85% of cases) and accounts for up to 80% of infection-related catheter loss. According to previous studies, *Staphylococcus aureus* nasal carriers have higher incidence of exit site infection and peritonitis rate then non-carriers. Therefore, nasal mupirocin prophylaxis is currently  recommended by the International Society for Peritoneal Dialysis (ISPD) Guidelines in 2005 for *S. aureus* prophylaxis. However, there are more and more reports of the emergence of mupirocin resistant *Staphylococcus aureus*, suggesting that eventually mupirocin will not be effective after long term intermittent use. Source control is a new idea for infection control. It has been proved in ICU patients | |
| 1. 試驗主題/背景：   全球約有15%尿毒症患者接受腹膜透析治療，其中，腹膜透析相關之感染併發症是導致患者治療失敗轉為接受血液透析或死亡的重要原因。常見的腹膜透析相關感染併發症包含有導管出口感染、隧道感染、或腹膜炎。依過去的研究資料顯示，鼻腔金黃色葡萄球菌(*Satphylococcus aureus*)帶菌之腹膜透析患者,相較於不帶菌之患者，有更高的比例會發生導管出口感染以及腹膜炎，因而，國際腹膜透析醫學會(ISPD)於2005年提出建議，針對鼻腔金黃色葡萄球菌帶菌之腹膜透析患者，可給予Mupirocin 2% oint塗抹於鼻腔治療，然而，長期使用Mupirocin後續引發之抗藥性問題目前仍令許多專家學者擔心。目前，有越來越多的證據顯示，利用感染源控制(Source Control)的方式，可預防住院患者發生院內感染，其中利用Chlohexidine幫助患者清潔洗澡已被證實具有良好感染源控制的效果。Chlohexidine消毒洗手液本身亦是國際腹膜透析醫學會所同意可用來做為導管出口照護之藥品，但目前尚無針對利用Chlohexidine消毒洗手液作為腹膜透析患者感染源控制之相關研究，故提出本研究計劃，希望比較加強鼻部及導管出口處感源控制之方式與傳統日常照護方式，兩者之間是否有具體降低腹膜透析患者感染之差異性。 | |
| 1. 試驗目的：欲達成之主要目的及其次要目的。   本研究希望藉由加強感染源控制來達成降低腹膜透析患者導管出口感染、隧道感染、以及腹膜炎之機會。  **主要研究目標**  將觀察比較有使用Chlohexidine消毒洗手液照顧傷口與一般照護方式之患者，從研究開始到第一次發生導管出口感染、隧道感染、或腹膜炎之時間有無差異。  **次要研究目標**  將觀察研究期間患者發生各種感染之時間、感染之菌種、因感染導致導管被迫移除之次數、以及對抗生素之抗藥性。 | |
| 四、試驗方法：包括 1.接受試驗者標準及數目 2.試驗設計及進行方法  3.試驗期限及進度 4.追蹤或復健計畫  5.評估及統計方法 | |
| 1. 受試者：   A.選擇標準：接受腹膜透析大於三個月以上，且可了解簽署受試者同意書之患者  B.排除標準：  排除標準:  1. 具精神疾病或無法確實配合傷口照護之患者  2. 最近一個月內有導管出口感染、隧道感染、或腹膜炎之患者  3. 最近一個月內有接受任何形式之抗生素治療患者  4. 對Mupirocin或Chlorhexidine過敏或無法接受之患者  受試者數目：預估100~120人  （2）試驗：  A.試驗設計：  a.進行方式：平行設計。  b.有對照組。  c.盲化方式：非盲。  d.隨機分派：使用隨機分派。同意參與研究之腹膜透析患者將先接受鼻部及導管出口處之細菌培養，再依帶菌狀況之有無，隨機分組。  B.進行方法：  患者將依隨機分派之方式分為治療組與對照組:  治療組: 若患者為Staphphylococcus aureus之nasal carrier，將先依照國際腹  膜透析醫學會(ISPD) 2005年之建議，給予Mupirocin 2% oint塗抹於鼻腔  治療，每天兩次共治療五天，此外，治療組之患者，每天導管出口清潔  換藥時，須使用4% Chlorhexidine藥水清潔傷口。  對照組: 若患者為Staphphylococcus aureus之nasal carrier，將先依照國際腹  膜透析醫學會(ISPD) 2005年之建議，給予Mupirocin 2% oint塗抹於鼻腔  治療，每天兩次共治療五天，此外，對照組之患者，每天導管出口清潔  換藥時，依照傳統模式，使用無菌之生理食鹽水清潔傷口。  臨床檢驗及觀察重點：受試者每月腹膜透析回診時須檢查導管出口之狀態並依據  國際腹膜透析醫學會(ISPD) 2005年之建議給予評分紀錄。此外，每月將重複鼻  部及導管出口處之細菌培養，以確定帶菌狀態並提供菌種及抗藥性分析。      （3）試驗期限及進度:  試驗期將維持18~24個月  **（4）評估及統計方法：**  **（6）主要療效或評估指標：**  **A.臨床觀察中用來作為主要療效或評估指標。**  **B.療效判定標準必須清楚界定，如增加多少為有效。**  **（7）統計方法**   1. **描述統計：著重於臨床上重要性的說明，例如描述預期治療效果或治療差異的幅度。**   **主要分析有接受Chlorohexidime 和沒有接受的兩組， 之後發生細菌移生的感染的機會是否不同。**  **單變數分析使用Chi-Square test，多變數分析，加入其他變數，包含年齡，性別，洗腎長久，是否最近住院使用抗生素等，進行Logistic regression，看是否有用Chloroxidemim此變數是獨立變數，預計使用SPSS10.-軟體進行分析**   1. **推論統計：檢定主要療效指標之統計顯著性所用之統計方法。單變數分析使用Chi-Square test，多變數進行Logistic regression。** 2. **期間分析：是否進行及進行時機。nil** | |
| 五、預期試驗效果 | |
| 預期實驗組患者之Staphylococcus aureus之nasal carrier以及導管出口處Staphylococcus aureus之colonization rate及re-colonization rate將會比對照組下降。此外，實驗組之患者的腹膜透析相關感染（導管出口感染、隧道感染、或腹膜炎）機會將比對照組明顯下降。 | |
| 六、可能傷害及處理 | |
| 可能傷害：  由於Chlorhexidine及Mupirocin皆為外用藥物，臨床副作用極少。目前，只有少數報告指出，在塗抹處皮膚可能會出現皮膚炎或過敏反應。  處理方式：  若患者出現皮膚不良反應，建議立即用無菌之生理食鹽水將藥物清洗掉，並立即回報緊急聯絡人，請患者盡快回診再視狀況給予抗過敏藥物或轉診皮膚科。  a. 回報時限：自受試者進入研究當日開始，至研究進行後二年以內。  b. 回報方式及程序：受試者有任何試驗相關問題可直接聯絡本試驗計劃主持人，或聯絡義大醫院之人體試驗委員會，請求諮詢。  c. 聯絡方法：試驗計劃主持人 王曦澔  人體試驗委員會 07-6150011轉分機5002 | |
| 七、所需藥品或儀器設備，包括必須進口之藥品或儀器名稱、數量。儀器並列明廠牌及型號。 | |
| Chlorhexidine gluconate 4% (Hibiscrub) 500ml/btl 約200瓶 (PBF)  Mupirocin 2% nasal ointment (Bactroban nasal ointment) 約200條 (GlaxoSmithKline) | |
